# Sequence Composition of Bacterial Chromosome Clones in a Transgressive Root-Knot Nematode Resistance Chromosome Region in Tetraploid Cotton

**DOI:** 10.3389/fpls.2020.574486

**Published:** 2020-12-14

**Authors:** Congli Wang, Mauricio Ulloa, Robert L. Nichols, Philip A. Roberts

**Affiliations:** ^1^Key Laboratory of Mollisols Agroecology, Northeast Institute of Geography and Agroecology, Chinese Academy of Sciences, Harbin, China; ^2^Department of Nematology, University of California, Riverside, Riverside, CA, United States; ^3^United States Department of Agriculture-Agricultural Research Service, Plains Area, Cropping Systems Research Laboratory, Plant Stress and Germplasm Development Research, Lubbock, TX, United States; ^4^Cotton Incorporated, Cary, NC, United States

**Keywords:** *Gossypium hirsutum*, *Meloidogyne incognita*, nematode resistance, NB-LRR, stress response elements, transposable elements

## Abstract

Plants evolve innate immunity including resistance genes to defend against pest and pathogen attack. Our previous studies in cotton (*Gossypium* spp.) revealed that one telomeric segment on chromosome (Chr) 11 in *G. hirsutum* cv. Acala NemX (*rkn1* locus) contributed to transgressive resistance to the plant parasitic nematode *Meloidogyne incognita*, but the highly homologous segment on homoeologous Chr 21 had no resistance contribution. To better understand the resistance mechanism, a bacterial chromosome (BAC) library of Acala N901 (Acala NemX resistance source) was used to select, sequence, and analyze BAC clones associated with SSR markers in the complex *rkn1* resistance region. Sequence alignment with the susceptible *G. hirsutum* cv. TM-1 genome indicated that 23 BACs mapped to TM-1-Chr11 and 18 BACs mapped to TM-1-Chr 21. Genetic and physical mapping confirmed less BAC sequence (53–84%) mapped with the TM-1 genome in the *rkn1* region on Chr 11 than to the homologous region (>89%) on Chr 21. A 3.1-cM genetic distance between the *rkn1* flanking markers CIR316 and CIR069 was mapped in a Pima S-7 × Acala NemX RIL population with a physical distance ∼1 Mbp in TM-1. NCBI Blast and Gene annotation indicated that both Chr 11 and Chr 21 harbor resistance gene-rich cluster regions, but more multiple homologous copies of Resistance (R) proteins and of adjacent transposable elements (TE) are present within Chr 11 than within Chr 21. (CC)-NB-LRR type R proteins were found in the *rkn1* region close to CIR316, and (TIR)-NB-LRR type R proteins were identified in another resistance rich region 10 cM from CIR 316 (∼3.1 Mbp in the TM-1 genome). The identified unique insertion/deletion in NB-ARC domain, different copies of LRR domain, multiple copies or duplication of R proteins, adjacent protein kinases, or TE in the *rkn1* region on Chr 11 might be major factors contributing to complex recombination and transgressive resistance.

## Introduction

With plant–pathogen co-evolution, plants have evolved two types of conserved innate immune systems, pathogen- or microbe-associated molecular pattern (PAMP/MAMP)-triggered immunity (PTI) (Monaghan and Zipfel, 2012) and effector-triggered immunity (ETI) (Dangl and Jones, 2001; Eitas and Dangl, 2010), to defend pathogen invasions. Pattern recognition receptors (e.g., receptor-like kinases or receptor-like proteins) localized in the plant cell surface detect microbial or pathogen structures (e.g., flagellins) to further activate PTI basal defense (Zipfel and Felix, 2005; Monaghan and Zipfel, 2012). Pathogenesis-related (PR) proteins are induced not only by phytopathogens but also by defense-related signaling molecules, such as salicylic acid (SA) and jasmonic acid (JA), ethylene (ET), and other phytohormones (Jones and Dangl, 2006). Based on the protein sequence, enzyme activities, and other biological characters, PR proteins are grouped into 17 families with various characters/functions, such as β-1,3-glucanases, peroxidase, proteinase inhibitor, thionin, oxidase-like, antifungal and antiviral, and so on (Sels et al., 2008). Plant resistance (*R*) genes are adapted to recognize specific pathogen avirulence (*avr*) genes to activate the secondary defense response (ETI) in species-specific disease resistance (Eitas and Dangl, 2010). ETI activation usually results in local cell death which is called hypersensitive response (HR). Resistance occurs when both the *R* gene and matching *avr* gene are present and disease occurs when either is inactive or absent (Flor, 1971). *R* genes encode five main classes of proteins (Dangl and Jones, 2001). Of these, intracellular nucleotide-binding site and carboxy-terminal leucine-rich repeat proteins (NBS-LRRs) are the largest class of *R* gene, and their function is more highly evolved and conserved (Eitas and Dangl, 2010). The binding of NBS with ATP or GTP functions as a signal transduction switch after the recognition of pathogen, and the LRR domain is involved in protein–protein interaction (Saraste et al., 1990; Dubey and Singh, 2018). The enlarged NBS domains are called NB-ARC (APAF-1, various R-protein and CED-4) domain, or Ap-ATPase domain, which contains additional homology between R proteins and effectors (van der Biezen and Jones, 1998; Aravind et al., 1999). The NBS-LRR class is subdivided as coiled-coil (CC)-NBS-LRR or Toll/inerleukin-1 receptor (TIR)-NBS-LRR based on N-terminal structural features that are required for downstream signaling following pathogen perception (Jones and Dangl, 2006; Qi and Innes, 2013). LRR is involved in auto-inhibition and/or effector detection (Kobe and Kajava, 2001). Furthermore, NBS-LRR receptors often contain repetitive sequences and transposable elements (TE) to form gene clusters (Meyers et al., 2003). TEs are considered as modulatory factors by insertion into the promoter region to regulate the nearby genes and thereby affect the epigenetic state (Slotkin and Martienssen, 2007; Deleris et al., 2016). In addition, stress-responsive genes or defense-related genes are modulated by DNA methylation and demethylation or histone modification (see reviews by Deleris et al., 2016; Espinas et al., 2016; Mauch-Mani et al., 2017; Tirnaz and Batley, 2019).

Among pathogens, plant parasitic nematodes utilize a stylet to penetrate roots, direct gland secretions into plants, and modify plant cells to develop more intimate and sophisticated modes of biotrophic parasitism (Williamson and Gleason, 2003). Root-knot nematodes (RKN, *Meloidogyne* spp.) and cyst nematodes (*Heterodera* and *Globodera*) are two major groups of economically important nematodes parasitizing a wide range of crop plants. Compared with bacterial, fungal, and virus resistance in plants, only seven nematode resistance genes have been cloned, *Hs1*^*pro–*^ from sugar beet, *Mi-1* and *Hero A* from tomato, *Gpa2* and *Gro1-4* from potato, and *rhg1* and *Rhg4* from soybean, which confer resistance to RKNs or cyst nematodes with different resistance structures (see review Williamson and Kumar, 2006; Cook et al., 2012, 2014; Liu et al., 2012). Of these, *Mi-1*, *Hero A*, *Gpa2*, and *Gro1-4* were classified into the NBS-LRR group of *R* genes and only *Gro1-4* contains a TIR domain (see review Williamson and Kumar, 2006). *Mi-1* confers resistance not only to several RKN species but also to insects (aphid and whitefly) (Williamson and Kumar, 2006). The potato cyst nematode resistance gene *Gpa2* shares a highly similar CC-NBS-LRR domain with potato virus X resistance gene *Rx1* in the same cluster region, and sequence exchange between homologous NBS-LRR genes converts the resistance specificity between nematode and virus (Slootweg et al., 2017). *Rhg1* and *Rhg4* resistance genes contain extracellular LRR motif and display resistance to various races of soybean cyst nematodes (see review Williamson and Kumar, 2006). The enzyme serine hydroxymethyltransferase (SHMT) encoded by *Rhg4* is associated with the resistance (Liu et al., 2012), and copy number of *rhg1* determines soybean resistance or susceptibility (Cook et al., 2012, 2014). Recently, Bayless et al. (2019) found a copia family retrotransposon was harbored within the *Rhg1*-encoded α-SNAP (alpha-soluble NSF attachment protein) gene in the *rhg1-a* (*Rhg1* low-copy) nematode resistance source.

In allotetraploid cotton (*Gossypium* spp.), southern RKN (*M. incognita*) is a major pathogen. A telomeric segment on chromosome (Chr) 11 was identified to contribute to *M. incognita* resistance from three major resistance sources, Acala N901 (NemX), Clevewilt 6, and Auburn 623 (Shen et al., 2006, 2010; Wang and Roberts, 2006; Wang et al., 2006; Ynturi et al., 2006; Gutiérrez et al., 2010; Roberts and Ulloa, 2010; Ulloa et al., 2010). Another resistance gene on Chr 14 originally derived from Wild Mexico Jack Jones was also identified to suppress nematode egg production and, when combined with the resistance gene on Chr 11 derived from Clevewilt, resulted in transgressive resistance in Auburn 623 (Gutiérrez et al., 2010; He et al., 2014; Kumar et al., 2016). In Acala NemX, a microsatellite (SSR) marker CIR316 tightly linked to the *rkn1* locus on Chr 11 contributes to both root-galling and nematode egg production resistance in the absence of the resistance on Chr 14 (Wang et al., 2006, 2017). However, little is known about the sequences and functions of the resistance genes. Wubben et al. (2019) identified one gene, Gh_D02G0276, at the Chr 14 locus that suppresses RKN egg production.

A transgressive factor, *RKN2*, on Chr 11 from highly susceptible *G. barbadense* cv. Pima S-7 combined with *rkn1* in Acala NemX produces transgressive resistance and mapped to the same region of the *rkn1* locus (Wang et al., 2008, 2017). In RIL F_2:7_ (Pima S-7 × Acala NemX), ∼40 centiMorgans (cM) of the telomeric region of Chr 11 contributed to resistance and fine mapping of the *rkn1* region with flanking markers CIR316 and CIR069 still does not conform as expected under Mendelian Law; 8 cM of the region accounted for more than 40% of the phenotypic variance with strong epistasis (Wang et al., 2017), thus revealing complex recombination in this region. Further, three-fold shorter genetic distance in the *rkn1* region on Chr 11 than that on Chr 21 in a testcross population explained 50–60% phenotypic variance, suggesting a cluster of genes work together for RKN resistance. Through quantitative trait loci (QTL) analysis of multiple segregating populations with the *rkn1* and *RKN2* loci, a three-gene model in the *rkn1* resistance region by tandemly arrayed allele (TAA) or gene (TAG) interactions was provided to explain the transgressive resistance (Wang et al., 2017). The *rkn1* region on Chr 11 from *G. barbadense* Pima 3-79 also contributed to transgressive resistance in a segregating population developed from two susceptible parents (*G. hirsutum* TM-1 × Pima 3-79) (Wang et al., 2012). The highly conserved homoeologous Chr 21 has no contribution to RKN resistance in Acala NemX (Wang et al., 2006, 2017) but has minor effects in other transgressive resistance sources (Wang et al., 2012). Both Chr 11 and Chr 21 harbor not only RKN resistance but also resistance to other multiple soil-borne diseases, including reniform nematode (*Rotylenchulus reniformis*) (Dighe et al., 2009; Gutiérrez et al., 2011), Fusarium wilt (*Fusarium oxysporum* f. sp. *vasinfectum*) (Ulloa et al., 2011, 2013, 2016; Wang et al., 2018), Verticillium wilt (*Verticillium dahliae*) (Bolek et al., 2005; Fang et al., 2014; Abdelraheem et al., 2020), and black root rot (*Thielaviopsis basicola*) (Niu et al., 2008).

Sequence composition of bacterial chromosome (BAC) clones from susceptible cv. Acala Maxxa and SSR markers on Chrs 11 and 21 revealed both chromosomes harbor resistance gene-rich genomic regions (Wang et al., 2012). Cotton whole genome sequence assemblies including diploid and tetraploid cultivars are available (Paterson et al., 2012; Li et al., 2014, 2015; Yuan et al., 2015; Zhang et al., 2015), and newly published genome comparisons provide insight into cotton A-genome evolution (Huang et al., 2020) and display genomic diversifications of five *Gossypium* allopolyploid species (Chen et al., 2020). However, these sequenced genomes are from susceptible genotypes, and the presence of transposable elements and multiple copies of NBS-LRR regions (Wang et al., 2015) make sequence assembly more difficult in the *rkn1* region. In an attempt to fine map the *rkn1* region by using the published genome sequence, only five more markers were mapped to that region compared with 21 markers on Chr 21 (Wang et al., 2017). The lack of polymorphic markers in the *rkn1* region confirms the complexity of the resistance. In addition, high similarity of the order of marker alleles amplified from the same primer pair was shown on Chr 11 and its homoeologous region of Chr 21 in various segregating populations (Wang et al., 2015, 2017) and highly conserved sequence between the two chromosomes (Paterson et al., 2012; Wang et al., 2012; Li et al., 2014, 2015; Zhang et al., 2015) indicate the unique structure and gene combination on Chr 11 that contributes to RKN resistance. Previously, we reported that a single nucleotide difference between Acala NemX and Acala SJ-2 results in phenotypic change for RKN resistance (Wang and Roberts, 2006). In order to provide further insight into the complex *rkn1* region, the resistant cultivar Acala N901 (Acala NemX) was used to make a BAC library, and the BAC clones around the Chr 11 *rkn1* region and its homoeologous Chr 21 region were identified and sequenced, and gene functions were annotated. Comparisons of BACs were conducted between Chr 11 and Chr 21 in Acala NemX and between resistant Acala NemX and susceptible Acala Maxxa (Wang et al., 2015).

## Materials and Methods

### Construction of Linkage Maps on Chr 11 and Chr 21 and Selection of SSR Markers for BAC Library Screening

A total of 395 polymorphic markers (Frelichowski et al., 2006; Wang et al., 2012, 2015, 2017; Yu et al., 2012) were used to screen 110 F_2:7_ (Pima S-7 × Acala NemX) RIL lines (Wang et al., 2017). The RKN resistance in Acala NemX was derived from Acala N901 (Goodell and Montez, 1994), which was used to make the BAC library. The genetic map was constructed with JoinMap^®^ version 4.0 program (Van Ooijen, 2006). The LOD threshold score > 4.0 and a maximum distance of 40 cM were used to determine linkage between any two markers. Originally, 45 SSR markers on Chr 11 and Chr 21 were chosen for BAC library screening. Most of these markers were in the region of *rkn1* or *RKN2* associated with RKN resistance on Chr 11 and its homoeologous region of Chr 21. SSR markers BNL3649, BNL1551, Gh132, BNL4011, and BNL3279 linked to reniform nematode resistance on Chr 21 (Gutiérrez et al., 2011) were also chosen for BAC screening.

### A Random Shear BAC Library Construction of *G. hirsutum* cv. Acala N901 (LuGnBAC) and the Identification of BAC Clones

Young leaves of *G. hirsutum* cv. N901 were collected and genomic DNA was randomly sheared into fragments of large inserts (>100 kb). A 5× coverage BAC library was constructed and cloned into the transcription-free pSMART BAC vector, and then, the vector was propagated in *Escherichia coli* DH10B by Lucigen (Lucigen, WI, United States). BAC clones were inoculated onto 384-deep-well library plates and grown individually. In total, the LuGnBAC had 158,208 clones arrayed in 412 384-well plates.

The LuGnBAC Library superpooling and pooling system had 34 SuperPool tubes and 34 corresponding Plate-Row-Column (P-R-C) pool collection plates (Lucigen). The first round PCR was performed on SuperPools to determine which SuperPool(s) contained the BAC clone(s) of interest. Based on the positive SuperPool(s), the second round PCR was performed on P-R-C plates to identify the exact plate and well position for the positive clone. The identified clone was used for sequencing and assembling. Through two-round PCR screenings, 64 BAC clones were confirmed to link to these 45 SSR markers, and each marker contained one–three BAC clones. The PCR amplification of cotton SSR markers was described by Ulloa et al. (2016).

### BAC Clone Culture and DNA Extraction and Purification

The identified BAC clones were picked to streak in fresh LB medium plates (Fisher Scientific) with 12.5 μg/ml chloramphenicol and the plates were cultured at 37°C overnight. A single colony from the overnight culture was inoculated into 2 ml fresh LB medium containing 10 g tryptone, 5 g yeast extract, and 10 g NaCl (Fisher Scientific) with 12.5 μg/ml chloramphenicol and grown with vigorous shaking (300 rpm) to late logarithmic phase (∼8 h) at 37°C. Then, 16 μl of the starter culture was added to 8 ml LB liquid medium in a larger volume vessel and grown with vigorous shaking at 300 rpm at 37°C overnight (16–18 h).

BAC DNA was extracted from the overnight liquid culture following the method of R.E.A.L. Prep 96 Plasmid kit (Qiagen). DNA yield was determined by Quant-iT^TM^ dsDNA Assay Kit (Invitrogen) with fluorescein (485 nm/535 nm) machine Wallac 1420 workstation (PerkinElmer, Inc., Turku, Finland). DNA quality was detected by using *HindIII* digestion of the BAC DNA at 37°C for over 2 h and checked on 1% agarose gel. Approximately 1–2.5 μg DNA was required for each clone sequencing.

### Sequencing and Assembly of Acala N901 (LuGnBAC) BAC Clones

The purified BAC DNA was sonicated to small fragments with high density between 300 and 400 bp by using Covaris S220 Ultrasonicator (Covaris, Woburn, MA, United States). The range of smear DNA was checked on 2% agarose gel. The fragmented DNA was end repaired, then dA-Tailed, and ligated with adaptor following the methods of NEBNext DNA Sample Prep Master Mix Set 1 (Biolab). The ligated DNA with the size range between 300 and 400 bp was selected on 2% agarose E-gel (Invitrogen). After PCR enrichment of the adaptor ligated DNA, the quantity and quality of each sample was checked by Agilent 2100 Bioanalyzer (Agilent). Sixty-four samples with different adaptor barcodes were sequenced with 2 × 100 bp paired-end read length in four flow cells of Illumina HiSeq 2000 system (Illumina) by the Genomics Center of University of California, Riverside.

The obtained sequences were subjected to de-multiplex analysis to separate each sample. All reads matching vector backbone and/or *E. coli* were removed with Bowtie software and default parameter and each sample was assembled with Velvet software. The consensus assembly was created with Cap3, using the program default parameters (Huang and Madan, 1999). These *de novo* contigs were then aligned to the TM-1 reference genome^1^ to aid the scaffold building. The gaps of less than 200 bp between the contigs aligned to the TM-1 genomes could be manually assembled together with N replacement. The same SSR markers with different clones were combined together after the alignment. The DNA sequence information of these BACs was deposited into GenBank with the accession numbers MW008609–MW008649. The BAC sequence assembly and the following gene annotation were conducted by Biomarker Technologies Co., LTD (Beijing, China).

### Annotation of BAC Sequence and Identification of Stress Response Elements

Bacterial chromosome sequences were analyzed first through Augustus (v.2.4) *de novo* gene prediction (Keller et al., 2011) and then GeMoMa (v1.3.1) homology-based prediction (Keilwagen et al., 2016, 2018) with reference genomes, including *G. hirsutum*, *G. raimondii*, *Arabidopsis thaliana*, and *Oryza sativa*. EVidenceModeler (EVM) (version 1.1.1) was used to combine the two gene prediction strategies to generate weighted consensus gene predictions (Haas et al., 2008). BAC sequences were blasted with *G. hirsutum* unigene set (GenBank release 165^2^) with an e-value ≤ 1e-5 and identity ≥ 90%. All predicted genes and unigenes were searched in a similar analysis with a value of 1e-5 using Blastx through the National Center for Biotechnology Information (NCBI)^3^ Nr (non-redundant) protein database to identify previously established protein motifs. The Blast2GO program with an e-value cut-off of 10^–5^ was utilized to classify the predicted GO (Gene Ontology) terms into three categories: biological process (BP), cellular component (CC), and molecular function (MF). Other public databases were also chosen for gene annotation, including Swiss-Prot^4^, COG (Clusters of Orthologous Groups^5^, KOG (Eukaryotic Ortholog Groups^5^), TrEMBL (Translations of the European Molecular Biology Laboratory nucleotide sequence entries^6^), and Pfam (Protein family^7^) with a similar e value cut-off of ≤1e-5. Stress response elements (SRE) were identified based on the description of gene annotation from these public databases. All the identified disease resistance proteins on Chr 11-Chr 21 BACs were aligned together with 4 Maxxa BACs [Chr 11-31K15-MUSB1076 (KM396697), Chr 21-AC190836-NAU6507, Chr 21-AC187810-NAU6525, and Chr 21-AC202830-NAU6697] containing resistance gene proteins (Wang et al., 2015).

### Alignment to *Gossypium raimondii* (D_5_), *G. arboreum* (A_2_), *G. barbadense* (AD2), and Other Genomes

In order to compare the BAC sequences from resistant Acala N901 with the reported genomes, the alignment of these BACs was conducted with the *G. raimondii* diploid D_5_ whole genome^8^ (Paterson et al., 2012), *G. arboreum* diploid A_2_ whole genome^9^ (Li et al., 2014), TM-1 AD_1_ genome (see text footnote 1), and Pima 3-79 AD_2_ genome^10^ (Yuan et al., 2015) through NCBI-nucleotide blast with an e-value ≤ 1e-10 and identity ≥ 90%. The BAC sequences on Chr 11 and Chr 21 were compared with corresponding chromosomes in A_2_, D_5_, AD_1_, and AD_2_ genome backgrounds. The consecutive matched sequences were chosen to compare these genomes, and the average identity and the percentage of mapped BAC sequences were calculated. Comparisons were also made between homologous BACs on Chr 11 and Chr 21 and between Acala Maxxa BACs and Acala N901 BACs that were associated with same SSR markers.

## Results

### BAC Sequencing, Assembly, and Alignment With the Reference TM-1 Genome

A total of 293M bp clear reads was obtained for all BACs and 98.79% Q30 were detected after quality filtering. After the same BAC clones or overlapped BACs were merged together, 41 BACs containing 45 SSR markers were reassembled with a total of 409 contigs, total contig length of 32.2M bp, N50 contig length of 35,123 bp (4,215–105,333 bp), N90 contig length of 16,439 bp (816–76,272 bp), ContigMax length of 40,069 bp (7,817–105,333 bp), and 32.5% GC content (Table 1). Aligned with the reference TM-1 genome (see text footnote 1), 24 markers in 23 BACs and 21 markers in 18 BACs were mapped to TM-1 Chr 11 and Chr 21, respectively (Table 1 and Supplementary Tables S1, S2). The average mapping identities (>99%), matched sequenced lengths, mismatches, gaps, and mapped ratios with the TM-1 genome are listed in Table 2. An 89.43% ratio of Chr 11 BACs and 89.34% of Chr 21 BACs aligned with the TM-1 genome were mapped to TM-1 Chr 11 and Chr 21, respectively (Table 2). Interestingly, only 53% of Chr11_6_UCR61 BAC sequences were mapped to the TM-1 genome, 71% for the BAC Chr11_5_UCR9, 80% for the nearby BAC Chr11_4_UCR102, and 84% for BAC Chr11_8_CIR316. On Chr 21 BACs, 40%, 42%, and 56% ratios were aligned to the TM-1 genome for Chr21_15_Gh132, Chr21_1_BNL2650, and Chr21_16_BNL1551, respectively (Table 2).

**TABLE 1:** The statistical filtered data and identified gene information for the assembled BACs on Chr 11 and Chr 21.

Chr 11	BACs	Contig number	Contig length	Contig N50	Contig N90	Contig Max	GC (%)	Gene number	Gene length
1	BNL2650	4	49,445	28,989	4,998	28,989	34.8	4	13,002
2	CIR069	6	58,873	31,139	4,293	31,141	30.8	2	4,600
3	UCR108	12	68,681	7,524	2,172	24,201	33.0	2	4,285
4	UCR102	3	48,126	29,461	4,859	29,461	30.2	2	6,576
5	UCR91	7	60,246	40,046	2,751	40,046	31.9	8	14,935
6	UCR61	61	171,322	6,707	1,397	28,505	33.2	12	23,264
7	MUSB1076	24	84,543	12,728	4,268	22,532	33.7	5	13,754
8	CIR316	5	99,691	28,435	4,245	37,380	32.7	9	35,774
9	UCR49	3	62,564	41,782	5,617	41,782	31.2	8	20,915
10	CM140-NAU1232	8	72,474	41,090	5,601	41,090	32.3	5	8,842
11	NAU2152	5	66,489	17,677	5,276	29,462	30.9	9	22,550
12	BNL1231	19	150,218	17,226	4,767	34,102	34.8	12	21,564
13	Gh288	19	71,620	5,780	2,475	7,817	30.6	9	20,461
14	BNL3279	21	72,307	12,145	4,727	24,944	34.2	10	20,073
15	CIR0003	9	76,697	31,055	6,782	32,152	31.2	3	10,509
16	NAU6507	18	60,505	8,948	1,048	14,030	33.8	6	13,109
17	NAU5505	2	84,350	73,662	10,688	73,662	31.4	11	31,820
18	Gh300	6	80,583	25,435	9,365	29,828	31.3	7	13,338
19	NAU4962	11	81,016	15,999	4,620	25,952	35.3	14	26,652
20	NAU3115	4	67,931	65,807	65,807	65,807	32.3	9	19,588
21	DPL0325	3	57,029	46,585	7,843	46,585	31.5	4	11,520
22	BNL1408	4	19,664	4,215	2,661	9,545	31.0	1	2,331
23	BNL3592	1	66,700	66,700	66,700	66,700	32.8	5	6,760
		255*	1,731,074*	28,658**	10,129**	34,161**	32.4**	157*	366,222*

**Chr 21**	**BACs**	**Contig number**	**Contig length**	**Contig N50**	**Contig N90**	**Contig Max**	**GC (%)**	**Gene number**	**Gene length**

1	BNL2650	2	63,929	59,780	59,780	59,780	35.7	15	24,909
2	DC40316	1	68,385	68,385	68,385	68,385	34.4	10	34,491
3	CIR069	9	53,966	15,314	2,997	20,682	33.1	0	0
4	GHACC1-UCR90	10	144,441	91,654	6,706	91,654	31.9	10	18,391
5	CIR316-UCR56	54	149,493	6,044	1,064	38,672	31.6	8	17,075
6	UCR49	7	101,127	33,436	25,236	36,288	31.0	9	12,756
7	NAU3480	2	68,109	53,885	14,224	53,885	31.8	5	17,089
8	MUCS088-CIR196	1	76,272	76,272	76,272	76,272	31.2	5	11,916
9	NAU5428	6	104,530	31,386	4,924	45,767	34.4	16	35,422
10	NAU6697	7	38,723	4,789	3,652	10,373	32.2	3	22,845
11	NAU2152	3	91,807	40,333	40,333	43,839	33.4	11	29,688
12	NAU6525	11	77,882	8,201	6,352	11,571	33.8	4	7,735
13	Gh288	3	72,660	38,968	28,193	38,968	32.1	7	31,359
14	BNL4011	19	51,688	29,227	816	29,227	30.8	0	0
15	Gh132	7	56,151	14,801	4,932	23,764	32.6	2	3,648
16	BNL1551	6	146,266	105,333	7,323	105,333	32.2	11	39,352
17	BNL3649	5	68,752	20,041	7,568	22,381	33.4	4	9,947
18	BNL1408	1	50,739	50,739	50,739	50,739	32.0	2	6,872
		154*	1,484,920*	41,588**	22,750**	45,977**	33.0**	122*	323,495*

*Total number. **Average number.

**TABLE 2:** Summary of Chr 11–Chr 21 mapping to the TM-1 genome (https://www.cottongen.org/analysis/189).

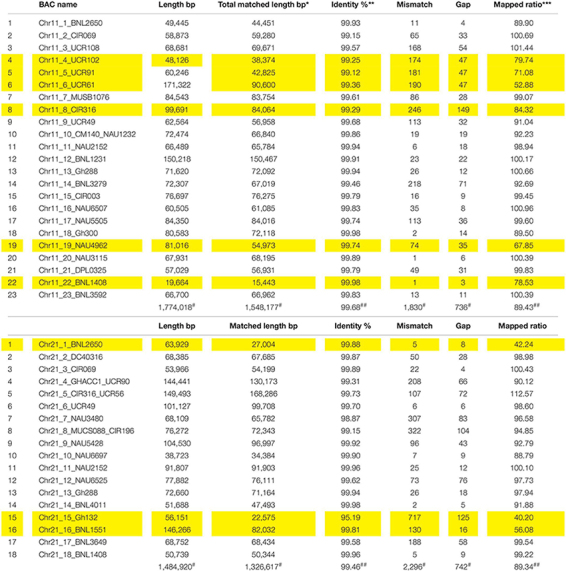

*Identity of the longest hit with TM-1 genome is shown if there are no consecutive match sequences found; with consecutive sequence, the average identity is shown only from the matched sequences. Please see Supplementary Tables S1, S2 for TM-1 AD genome comparison. **Total length of continuous sequence on the same region of TM-1 genome. ***Mapped ratio equal mapped sequence length/BAC length. ^#^Total number. ^##^Average number. The highlight in yellow indicates that the mapped ratio of BACs to TM-1 genome is less than 85%.

**FIGURE 1:**
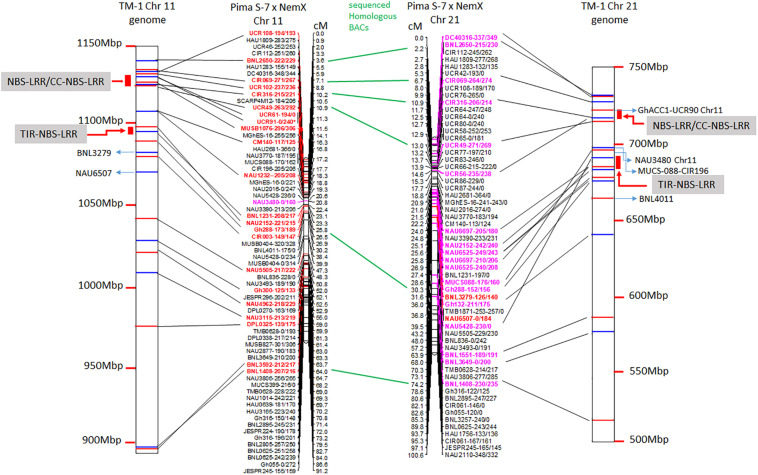
Alignment of TM-1 reference genome (https://www.cottongen.org/analysis/189) with linkage maps of Chr 11 and its homoeologous Chr 21 using an interspecific [Pima S-7 (*Gossypium barbadense*) × Upland Acala NemX (*G. hirsutum*)] RIL population (Wang et al., 2017). SSR markers in red are mapped to TM-1 Chr 11 genome and those in pink are mapped to TM-1 Chr 21 genome.

The linkage maps derived from the RIL F_2:7_ Pima S-7 × Acala NemX population were constructed through screening 395 polymorphic markers (Wang et al., 2017), and 66 and 59 markers were mapped to Chr 11 and its homoeologous Chr 21, respectively (Figure 1). The physical map of these BACs on the TM-1 genome was aligned with the linkage map (Figure 1). The genetic distance between CIR316 and CIR069 is 3.1 cM in the RIL population and the physical distance is 1 Mbp in the TM-1 genome (Figure 1). The relocation of the markers within Chr 11 or Chr 21 and between Chr 11 and Chr 21 was observed in the genetic map and TM-1 physical map (Figure 1). For example, SSR markers UCR108, UCR49, UCR61, UCR91, and MUSB1076 were mapped outside of the interval flanked by markers CIR069 and CIR316 in the RIL population but their associated BACs were mapped to a 1-Mbp segment in the TM-1 genome within the interval between markers CIR069 and CIR316 (Figure 1 and Supplementary Table S1). The SSR markers GhACC1-UCR90, NAU3480, and CIR196 were mapped to Chr 11 in the F_2:7_ (Pima S-7 × Acala NemX) RIL population (Figure 1) (Wang et al., 2017), but the associated BACs were mapped to Chr 21 in the TM-1 genome (Figure 1). Similarly, BNL3279 and NAU6507 were mapped to Chr 21 in the F_2:7_ RIL population, but their BACs mapped to Chr 11 in the TM-1 physical map (Figure 1). All the BAC sequences were deposited in the NCBI database with accession numbers MW008609–MW008649.

### BAC Sequence Blasted With *Gossypium hirsutum* Unigene Set

**FIGURE 2:**
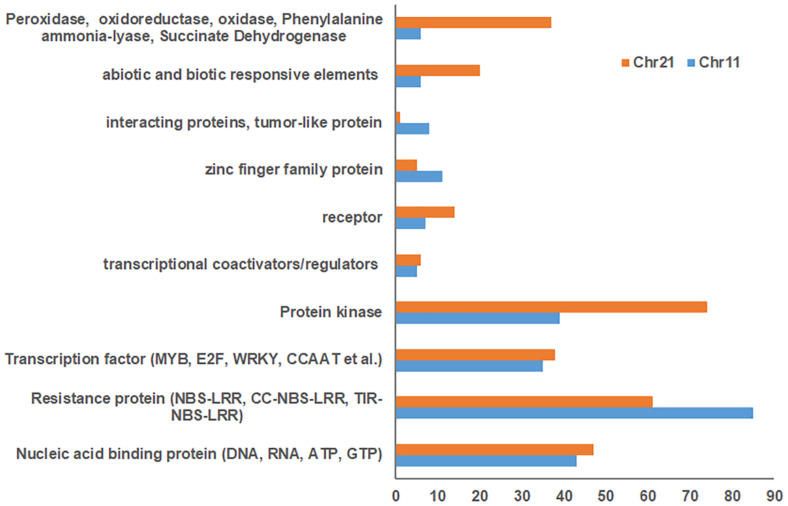
Comparison of number of stress response elements on Chr 11 BACs with those on Chr 21 BACs based on *Gossypium hirsutum* Unigene NCBI database.

Blasting with the *G. hirsutum* Unigene set indicated 1,832 and 1,930 unigenes for Chr 11 and Chr 21 BACs, respectively (Supplementary Tables S3, S4). Of these, 796 uncharacterized proteins were found on Chr 11 BACs and 865 on Chr 21 BACs. In addition, 492, 504, 234, and 152 unigenes on Chr 11 BACs and 508, 495, 256, and 174 unigenes on Chr 21 BACs had homology with *Populus trichocarpa*, *Vitis vinifera*, *Ricinus communis*, and *Arabidopsis thaliana*, respectively (Supplementary Tables S3, S4). The stress response elements on these BACs were classified as resistance proteins (e.g., NBS-LRR, RPP8, RPP13, and RGA2) (Chr 11/Chr 21, 85:61), nucleic acid binding protein, transcription factors (MYB, E2F, WRKY, CCAAT, etc.), protein kinase (Chr 11/Chr 21, 39:74), receptor, tumor-like proteins, abiotic and biotic responsive elements (Chr 11/Chr 21, 6:20), plus others (Figure 2 and Supplementary Tables S5, S6). Interestingly, the markers UCR61, CIR316, and MUSB1076 associated with *rkn1* regions contained NBS-LRR/CC-NBS-LRR-type resistance proteins, and the markers BNL1231 and NAU2152, further away from the *rkn1* locus, contained TIR-NBS-LRR-type resistance proteins. Surprisingly, the BAC associated with another important marker CIR069 linked to a RKN resistance locus on Chr 11 in resistant *G. hirsutum* cv. M120 (Shen et al., 2006) had no R proteins but only serine/threonine protein kinase. Similarly, the marker CIR316 region on Chr 21 contained (CC)-NBS-LRR proteins and the NAU2152 region contained TIR-NBS-LRR proteins. Of these, the BAC Chr-11_6_ UCR 61 contained 54 NBS-LRR unigenes, BAC Chr11_8_CIR316 with 9 NBS-LRR unigenes, and BAC Chr11_12_BNL1231 with 14 NBS-LRR unigenes (Supplementary Tables S3, S5). The BAC Chr21_5_CIR316-UCR 56 contained 23 NBS-LRR unigenes and BAC Chr21_12_NAU6525 close to the NAU2152 region had 21 NBS-LRR unigenes (Supplementary Tables S4,S6).

### BAC Gene Annotation and Stress Response Elements

Augustus (v.2.4) *de novo* gene prediction followed with GeMoMa (v1.3.1) homology-based prediction indicated that the length of 157 genes ranged from 2,331 to 31,820 bp on Chr 11 and the length of 122 genes ranged from 7,735 to 39,352 bp on Chr 21 (Table 1). The gene names, coding sequence (cds), and encoded amino acids (protein) are listed in Supplementary Tables S7–S12. The gene annotation with GO, GO summary tree, Nr, Swiss-Prot, COG, KOG, TrEMBL, and Pfam are given in Supplementary Tables S13–S20 for Chr 11 and Supplementary Tables S21–S28 for Chr 21. The resistance proteins, transcription factors, receptors, and associated stress response elements are highlighted in yellow and transposable elements in red. In the Pfam description, 15 disease resistance proteins, 16 other stress-responsive elements (transcription factors, protein kinase, receptors, etc.), and 32 transposable elements (gag-polypeptide) and relative reverse transcriptase were identified on Chr 11 BACs (Table 3). Nine disease resistance proteins, 21 other stress responsive elements, and 10 transposable elements were classified on Chr 21 BACs (Table 4). Interestingly, there were two genes on the Chr 11-CIR069 BAC but none on the Chr 21-CIR069 BAC.

**TABLE 3:** The Pfam (Protein family, http://pfam.xfam.org/) gene annotation of Chr 11 BACs with a similar *e* value cutoff ≤ 1e-5.

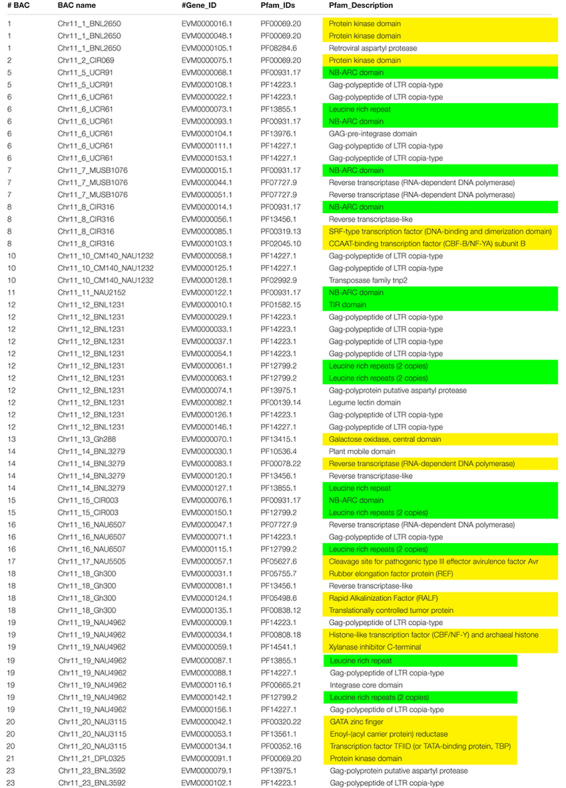

*Disease resistance proteins are highlighted in green; transcription factors, protein kinase, and other stress-responsive elements in yellow; and transposable elements or relative reverse transcriptase remain black letters with white background.*

**TABLE 4:** The Pfam (Protein family, http://pfam.xfam.org/) gene annotation of Chr 21 BACs with a similar *e* value cutoff ≤ 1e-5.

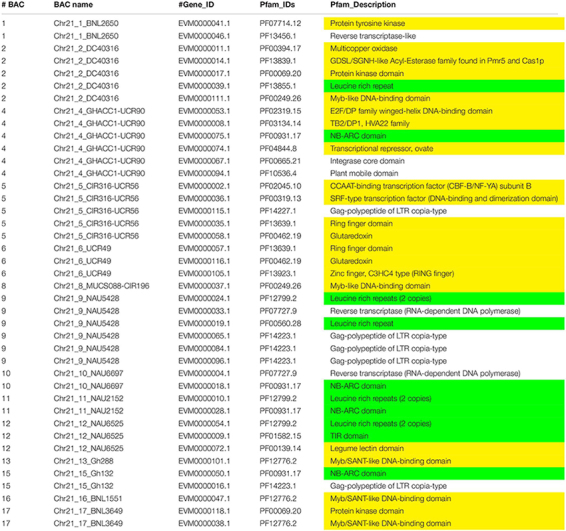

*Disease resistance proteins are highlighted in green; transcription factors, protein kinase, and other stress-responsive elements in yellow; and transposable elements or relative reverse transcriptase remain black letters with white background.*

### Sequence Alignments of Disease Resistance Proteins, Transcription Factors, or Transposable Elements Among Acala N901 Chr 11-Chr 21 BACs and Maxxa BACs

**FIGURE 3:**
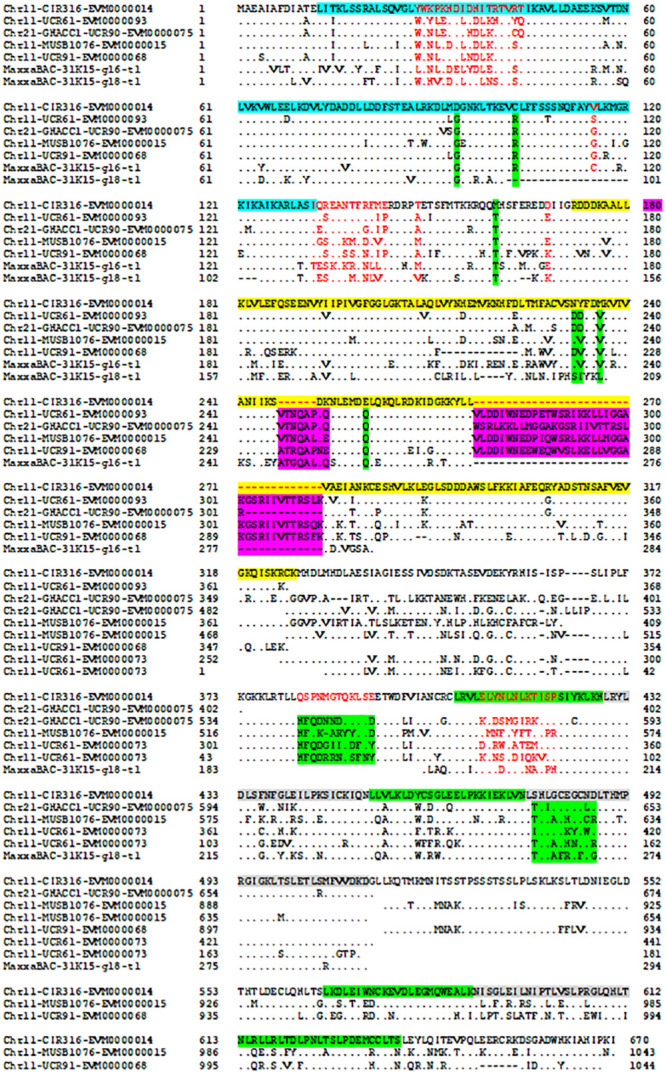
Alignment of disease resistance protein (CC)-NBS-LRR among BACs on Acala N901 Chr11-21 BACs and Acala Maxxa BAC 31K15 (Wang et al., 2015) in the *rkn1* region. The sequence in light blue in the first line represents CC domain, with NB-ARC domain in yellow and LRR domains in green and gray.

The protein–protein Blast aligned the identity of the homologous regions of disease resistance proteins. The resistance proteins in BACs associated with the markers UCR91, UCR61, MUSB1076, and CIR316 on Chr 11 are homologous and contain (CC)-NBS-LRR motif (Figures 1, 3). These proteins are also homologous with Acala N901 BAC Chr21_4_GHACC1_UCR90 and Acala Maxxa BAC 31K15 associated with MUSB1076 markers (31K15:g16.t1; 31K15:g18.t1) (Wang et al., 2015). Multiple homologous copies are present in the sequences (Figure 3) and 48–79% identities were found with substitution or insertion/deletion. The disease resistance proteins with (TIR)-NBS-LRR motif in BAC Chr11_11_NAU2152 are homologous with Chr11_12_BNL1231, Chr21_9_NAU5428, Chr21_10_NAU6697, Maxxa BACs AC202830 (linked to Chr 21 SSR marker NAU6697), and AC187810 (linked to Chr 21 SSR marker NAU6525) (Supplementary Figure S1). The identities among these BACs were up to 68–75%.

The encoded protein of the gene EVM0000068.1 in BAC Chr11_5_UCR91 shares 97% identity with putative disease resistance protein RGA4 (*G. arboreum*) (NCBI: XP_017630309.1) and 74% identity with disease resistance protein RGA2-like isoform X1 (*G. raimondii*) (NCBI: XP_012435026.1) (Supplementary Figure S2). The CC-NB-ARC and LRR domains of the gene EVM0000014.1 in the BAC Chr11_8_CIR316 share 75% and 69% identity with the same domains of RGA2-like isoform X1 (NCBI: XP_012435026.1), respectively (Supplementary Figure S3). The 120 amino acids (aa) at the 13–132 aa location have homology with Rx-CC-like domain of potato virus X resistance protein and similar proteins (Figure 3, highlighted in light blue). Interestingly, six aa between the position 247 and 248 and 37 aa between the position 270 and 271 are always missing in the NB-ARC region (highlighted in yellow, Figure 3) of the gene EVM0000014.1 compared with other homologous resistance proteins (Figure 3 and NCBI database). In addition, the seven LRR domains between aa 407 and aa 670 (highlighted in green and gray) in the gene EVM0000014.1 were found homologous with RGA2-like isoform X1 (*G. raimondii*) (NCBI: XP_012435026.1) that contains 11 LRR domains. The TIR-NBS-LRR protein of resistance gene EVM0000122 in BAC Chr_11_NAU2152 shares 95% identity with TMV resistance protein N-like (*G. hirsutum*) (XP_016755134.1), and in NB-ARC domain, 22 aa are missing between the position 342 and 343 aa compared with all reported TMV resistance proteins N-like and other compared sequences in this study (highlighted in yellow, Supplementary Figure S4). There are eight LRR regions located between aa 548 and aa 833. The phylogenetic tree for these R protein sequences on both Chr 11 and Chr 21 is presented in Supplementary Figure S5.

In addition, identities of 86–93% for the alignments of CCAAT-binding transcription factor CBF-B/NF-YA (86%), SRF (serum response factor)-type (93%) transcription factor (DNA-binding and dimerization domain), and protein kinases (90–91%) were detected between BAC genes on Chr 11 and Chr 21, which are adjacent to the *rkn1* resistance region (Supplementary Figures S6–S9). Substitutions and insertions/deletions were found in the alignments (Supplementary Figures S6–S9). Interestingly, there are 18 aa (VNAKQYKGIMRRRQSRAK) missing in the location between aa 58 and aa 59 in the CBF-B/NF-YA subunit B in the gene EVM0000103 on BAC Chr11_8_CIR316 compared with the same transcription factor on BAC Chr 21_5_CIR316_UCR56 (Supplementary Figure S2) and other reported homologous sequences, such as nuclear transcription factor Y subunit A-10 in *Gossypium* spp. and other genomes (NCBI Blast database).

Thirty-two TEs and relative reverse transcriptase were identified on Chr 11 (Table 3) compared to 10 on Chr 21 (Table 4). The coding sequence comparisons of transposable elements and relative reverse transcriptase indicated 80–99% identity (Supplementary Table S29) among Acala N901 BACs (Table 1) and 4 Maxxa BACs (31K15, AC190836, AC202830, and AC187810) with high resistance gene analog (RGA) content (Wang et al., 2015). Interestingly, the TEs with highly similar sequences always occurred in certain BACs full of RGA, such as in the regions of CIR 316 and BNL1231 (Supplementary Table S29 and Tables 3, 4). The high homology was also found within Acala N901 Chr 11-BACs (Supplementary Table S29), indicating multiple copies of TEs in the *rkn1* region on Chr 11.

### Alignment to *G. arboreum* (A_2_), *G. raimondii* (D_5_), and *G. barbadense* (AD2) Genomes

The alignment of Chr 11–21 BACs to the A2, D5, and AD2 genomes revealed that the similarity among the genomes was different and that different BAC orders were found in the compared data (Table 5 and Supplementary Tables S30–S35). The Chr 11 BACs had greater alignment to the A2 genome than to D5 and AD2. The Chr 21 BACs had greater alignment to the D5 genome than to the other two genomes (Table 5).

**TABLE 5:** Acala N901 BAC alignment to *Gossypium arboreum* (A_2_), *G. raimondii* (D_5_), *G. barbadense* (AD2).

	*%	**Total		Gap		%	Total		Gap
Chr11	identity	alignment	Mismatches	opening	Chr21	identity	alignment	Mismatches	opening
A2	96.382	1,014,693	19,456	3,189	A2	94.115	781,143	31,873	4,845
D5	93.192	615,953	27,896	4,718	D5	95.006	1,249,918	29,757	5,686
AD2	97.320	537,583	5,694	1,001	AD2	96.218	425,846	6,549	1,106

*The average identity is shown only from the matched sequences. Please see Supplementary Tables S30–S35. **Total length of continuous sequence on the same region of the compared genome.

## Discussion

This study represents the first time sequence comparisons between Chr 11 and its homoeologous Chr 21 were made in RKN resistant line Acala N901 BACs from which the *rkn1* locus in Acala NemX was first identified (Wang et al., 2006). The physical and genetic mapping confirmed both Chr 11 and Chr 21 had high sequence homology; however, less mapped sequence with RKN susceptible TM-1 was identified in the *rkn1* region linked to SSR markers CIR316, UCR 61, and UCR 91 on Chr 11 than that on Chr 21, which might explain the differences in resistance between the pair of chromosomes. NCBI Blast and Gene annotation indicated that both Chr 11 and Chr 21 harbor resistance gene-rich regions (Figure 1), but with more resistance proteins on Chr 11 than on Chr 21 (Figure 2 and Tables 3, 4). More multiple homologous copies of R proteins and adjacent TE are present within Chr 11 than within Chr 21, and insertion/deletion and substitution in the protein domain were found among these homologous R proteins and TE on both Chr 11 and Chr 21, indicating transposable elements might be involved in RKN resistance and the transgressive resistance in the *rkn1* region. Furthermore, the change of one nucleotide in the *rkn1* region could alter the phenotype from susceptible to resistant as previously reported (Wang and Roberts, 2006). The further functional characterization of these R protein domains will reveal the transgressive resistance mechanism, shed light on resistance evolution, and be helpful for developing new sources of resistant crops.

It is well known that the NB-ARC domain is a functional ATPase domain which can regulate R protein activity (van Ooijen et al., 2008). The KOG annotation revealed six genes (EVM0000014, EVM0000015, EVM0000068, EVM0000073, EVM0000076, and EVM0000093) contain an ATPase domain which regulates apoptotic cell death (Supplementary Table S18). Interestingly, NCBI blast results indicated two deletions in the (CC)-NB-ARC domain for the resistance gene EVM0000014.1 in the BAC Chr11_8_CIR316 and one deletion in the (TIR)-NB-ARC domain for the resistance gene EVM0000122 in BAC Chr_11_NAU2152, compared with other R genes on both Chr 11 and Chr 21 in this study and with other reported resistance genes in *Gossypium* spp. and other species. The gene EVM0000014.1 associated with CIR316 is closely linked to the *rkn1* locus and the gene EVM0000122 associated with NAU2152 adjacent to BNL1231 is another important RKN resistance region (Wang et al., 2006, 2008, 2017). The NB-ARC domain contains three conserved ATP/GTP binding motifs (kinase-1a or P-loop, kinase-2, and kinase-3a) which are critical for nucleotide binding (Traut, 1994). The mutations of motif residues cause either loss-of-function or auto-activation of the NB-LRR protein (Tameling et al., 2002; van Ooijen et al., 2008). Tameling et al. (2002) confirmed that a functional nucleotide binding pocket is formed in the NBS domain through mutation studies of the NB-ARC domain in the R proteins I-2 conferring resistance to *Fusarium oxysporum* and Mi-1 conferring resistance to RKN and potato aphids. Mutation in P-loop reduces ATP binding capacity (Tameling et al., 2002), and mutations in ATP binding or ATP hydrolysis indicated that ATP hydrolysis is not necessary for signaling initiation but it is very important to maintain R-protein activity in the absence of plant pathogens (auto-activation) (Tameling et al., 2006). The LRR domain can physically interact with the NB-ARC domain, and deletions of LRR domain result in auto-activation (Qi et al., 2012; Qi and Innes, 2013). The deletion/insertion of the NB-LRR domain or varying numbers of LRRs in cotton may underlie the unique structure in the *rkn1* regions that results in RKN resistance determination on Chr 11 but neither on Chr 21 (Wang et al., 2006, 2017) nor in other susceptible genomes.

Multiple copies of LRR proteins among the markers CIR316, UCR91, UCR61, and MUSB1076 linked to the *rkn1* resistance locus on Chr 11 and UCR90 on Chr 21 (Figures 1, 3) could result in complex recombination in the *rkn1* region when combined through crossing with other genomes, and consequently, the R gene regions cannot be separated just based on phenotypes. This phenomenon was observed in different segregating populations with Acala NemX as one of the parental lines (Wang et al., 2017). A three-fold shorter interval in the clustered *rkn1* region on Chr 11 than that on Chr 21 in a NemX × F_1_ (Pima S-7 × SJ-2) testcross population (Wang et al., 2017) indicated that complex recombination might result from the multiple copies of R proteins in the *rkn1* region. The relocation of markers UCR61, UCR91, and MUSB1076 in an F_2:7_ (Pima S-7 × NemX) population is beyond the range of CIR316 and CIR069 (Figure 1), the two important SSR markers linked to RKN resistance (Shen et al., 2006; Wang et al., 2006), but they are re-localized between markers CIR316 and CIR069 in the TM-1 physical map (Figure 1), suggesting R gene relocation between susceptible and resistant cotton genotypes possibly by duplication and recombination of R genes (Leister, 2004). That the CIR069-BAC contains no R protein but has a kinase protein suggests that the *rkn1* gene might be close to CIR316 which contains R proteins in Acala NemX. Further, in comparing the genetic mapping and physical mapping results, the exchange of SSR markers (e.g., GhACC1-UCR90, CIR196, BNL3279, and NAU6507) between the homoeologous chromosome pair might provide more clues for resistance evolution between R and S parental lines.

The CCAAT-binding transcription factor (CBF-B/NF-YA) close to R genes associated with CIR316 BAC on Chr 11 had an 18-aa deletion compared with Chr 21 and other genomes, indicating unique structure in this region, too. The conserved CCAAT-binding factor is present in the promoter and is important for transcription (Mantovani, 1998, 1999), and NF-YA is necessary for DNA binding (Sinha et al., 1995). The CCAAT-binding factor plays a direct role in regulating iron uptake/utilization and the oxidative stress response (Chakravarti et al., 2017). Hu et al. (2019) identified 10,366 genes with sequence variations (GSV) between *G. hirsutum* TM-1 and *G. barbadense* Hai7124, and these GSVs result in gain or loss of stop codons and frameshifts and consequently cause phenotypic divergence. A 2-bp (CA) deletion in *WLIM1a* involved in cotton fiber elongation and secondary cell wall formation in Hai7124 results in premature termination of transition compared with that in TM-1 (Hu et al., 2019). The species-specific Indel was common in cotton (Hu et al., 2019).

More transposable elements and greater multiple copies of TE adjacent to R proteins in the *rkn1* region were identified on Chr 11 than on Chr 21 (Tables 3, 4), suggesting TE might be involved in resistance versus susceptibility. TEs as mobile genetic elements are major drivers of plant genome evolution by facilitating gene mutation, duplication, movement, and novel gene creation. TEs are classified into retrotransposons and DNA transposons. Retrotransposons include long-term repeat (LTR) retrotransposons (*Ty1*/*Copia* and *Ty3*/*Gypsy*) and non-LTR retrotransposons (Wicker et al., 2007). TEs represent ∼60% of the genome in *G. hirsutum*, *G. raimondii*, and *G. barbadense* (Paterson et al., 2012; Zhang et al., 2015; Hu et al., 2019). In the *G. raimondii* genome, 14,332 TEs were structurally annotated and clearly categorized into seven distinct super-families, with 2,929 *copia-like* elements and 10,368 *Gypsy-like* elements (Xu et al., 2017). In this study, *copia-like* elements were found nearby R protein genes. Almost two times more TEs in the A sub-genome than the D sub-genome were reported in *G. hirsutum* cv TM-1 genome and *G. barbadense* cv. Hai7124 (Zhang et al., 2015; Hu et al., 2019), and unsurprisingly, the diploid *G. arboreum* A genome has 2.66× more TEs than the diploid *G. raimondii* D genome (Li et al., 2014). That polyploidization can induce TE activity (Vicient and Casacuberta, 2017) might explain the abundance of TEs in *G. hirsutum*. Chen et al. (2020) compared five allotetraploid genomes and found that TE exchanges between A and D subgenomes make genome-size equilibration following allopolyploidy. TEs are often localized nearby protein-coding genes or in their introns to control gene expression in various ways. TE regulatory functions work as *cis*-regulatory networks to affect nearby gene expression, modification of the chromatin state of gene promoters, post-transcriptional levels, and epigenetic silencing of the host genome to control gene expression (siRNA) (Vicient and Casacuberta, 2017). In response to stress, TE activation and TE modification of gene expression play important roles to enable plants to adapt to stress conditions (Ito et al., 2011, 2016; Barah et al., 2013; Cowley and Oakey, 2013; Cavrak et al., 2014; Makarevitch et al., 2015). TE-derived siRNA can also regulate the stress response (McCue et al., 2012). TEs regulating defense or susceptibility genes also have been reported (Tsuchiya and Eulgem, 2013; Berg et al., 2015). An intact *copia* retrotransposon (*RAC*) was found within intron 1, anti-sense to *H. glycines* resistance gene *rhg1-a α-SNAP* open reading frame, and RAC has intrinsic promoter activities in soybean (Bayless et al., 2019). Hu et al. (2019) identified the unevenly distributed presence/absence variants of gene structure across the genome between TM-1 and Hai7124 to be mostly *copia* elements (LTR type) that were found in coding DNA sequence. The unexpected structure features of resistance genes resulting from TE movement are no doubt involved in deviations from expected Mendelian Law, as we reported previously for RKN resistance in cotton (Wang et al., 2017). In this study, the abundant TEs found adjacent to *R* genes in the resistance gene cluster regions on Chr 11 and comparisons of sequence information between the homoeologous pair of chromosomes 11 and 21, and between resistant and susceptible genotypes, shed more light on the transgressive resistance mechanism in cotton.

## Data Availability Statement

The DNA sequence information of these BACs was deposited into GenBank with the accession numbers MW008609–MW008649.

## Author Contributions

PR, CW, MU, and RN conceived and designed the study. CW performed the laboratory work and conducted data analysis. CW wrote the original draft. PR, MU, and RN reviewed and edited the manuscript. All the authors read and approved the final manuscript.

## Conflict of Interest

RN was employed by the company Cotton Incorporated. The remaining authors declare that the research was conducted in the absence of any commercial or financial relationships that could be construed as a potential conflict of interest.

## Footnotes

1 https://www.cottongen.org/analysis/189

2 http://www.plantgdb.org

3 http://www.ncbi.nlm.nih.gov/

4 http://www.uniprot.org/

5 http://www.ncbi.nlm.nih.gov/COG

6 http://www.bioinfo.pte.hu/more/TrEMBL.htm

7 http://pfam.xfam.org/

8 http://phytozome.net

9 https://www.cottongen.org/species/Gossypium_arboreum/bgi-A2_genome_v2.0

10 https://www.cottongen.org/species/Gossypium_barbadense/nbi-AD2_genome_v1.0

## Supplementary Material

Supplementary Figure 1 Alignment of disease resistance protein (TIR)-NBS-LRR among Acala N901 Chr 11–21 BACs and Acala Maxxa BACs (31k15, AC187810, AC202830). The sequence in light blue in the first line represents CC domain, NB-ARC domain in yellow, and LRR domains in green and gray.

Supplementary Figure 2 Comparison of (CC)-NB-LRR protein of Chr11-UCR91- EVM0000068.1 with RGA4 (*Gossypium arboreum*, XP_017630309.1) and RGA2-like isoform X1 (*Gossypium raimondii*, XP_012435026.1) in cotton.

Supplementary Figure 3 Comparison of (CC)-NB-LRR protein of Chr11-UCR316-EVM0000014.1 with RGA2-like isoform X1 (*Gossypium raimondii*, XP_012435026.1) in cotton.

Supplementary Figure 4 Comparison of (TIR)-NB-LRR protein of Chr11-NAU2152-EVM0000122.1 with TMV resistance protein N-like in cotton. The sequence in light blue in the first line represents CC domain, NB-ARC domain in yellow, and LRR domains in green and gray.

Supplementary Figure 5 The evolutionary tree of amino acid sequences of *R* genes on Chr 11 and Chr 21.

Supplementary Figure 6 Comparison of CCAAT-binding transcription factor (CBF-B/NF-YA) subunit B in the *rkn1* region between Chr 11-CIR316 and Chr 21-CIR316.

Supplementary Figure 7 Comparison of SRF-type transcription factor (DNA-binding and dimerisation domain) in the *rkn1* region between Chr 11-CIR316 and Chr 21-CIR316-UCR56.

Supplementary Figure 8 Comparison of protein kinase domain in the *rkn1* region between Chr 11-BNL2650 and Chr 21- BNL2650.

Supplementary Figure 9 Comparison of protein kinase domain in the *rkn1* region between Chr 11-BNL2650 and Chr 21-DC4031.

Supplementary Tables 1, 2 Chr11 (S1) and Chr 21 (S2) BAC mapping to *Gossypium hirsutum* TM-1 genome (https://www.cottongen.org/analysis/189).,

Supplementary Tables 3–6 *Gossypium hirsutum* Unigenes of Acala N901 BACs on Chr 11 (S3) and Chr 21 (S4) BACs and stress response elements of *G. hirsutum* Unigenes on Chr 11 (S5) and Chr 21 (S6) BACs.

Supplementary Tables 7–12 Predicted genes, coding sequences (CDS), and peptides on Chr 11 (S7–S9) and Chr 21 (S10–12) BACs.

Supplementary Tables 13–20 Gene annotation on Chr 11 BACs, including GO (S13), GO summary tree (S14), Nr (S15), SwissProt (S16), COG (S17), KOG (S18), TrEMBL (S19), and Pfam (S20).

Supplementary Tables 21–28 Gene annotation on Chr 21 BACs, including GO (S21), GO summary tree (S22), Nr (S23), SwissProt (S24), COG (S25), KOG (S26), TrEMBL (S27), and Pfam (S28).

Supplementary Table 29 The coding sequence comparisons of transposable elements and relative reverse transcriptase among N901 BACs and 4 Maxxa BACs (31K15, AC190836, AC202830, AC187810).

Supplementary Tables 30–32 The alignment of Chr 11 BACs to *Gossypium arboreum* A2 (S30), *G. raimondii* D5 (S31), and *G. barbadense* AD2 (S32).

Supplementary Tables 33–35 The alignment of Chr 21 BACs to *Gossypium arboreum* A2 (S33), *G. raimondii* D5 (S34), and *G. barbadense* AD2 (S35).
