# Supplementary material for: Sequence Composition of Bacterial Chromosome Clones in a Transgressive Root-Knot Nematode Resistance Chromosome Region in Tetraploid Cotton
Source: Front Plant Sci. 2020 Dec 14;11:574486. doi: 10.3389/fpls.2020.574486 (PMC7767830; doi:10.3389/fpls.2020.574486)
Supplement: Supplementary Figure 1 — Alignment of disease resistance protein (TIR)-NBS-LRR among Acala N901 Chr 11–21 BACs and Acala Maxxa BACs (31k15, AC187810, AC202830). The sequence in light blue in the first line represents CC domain, NB-ARC domain in yellow, and LRR domains in green and gray. [file Presentation_1.PPTX]

## Slide 1
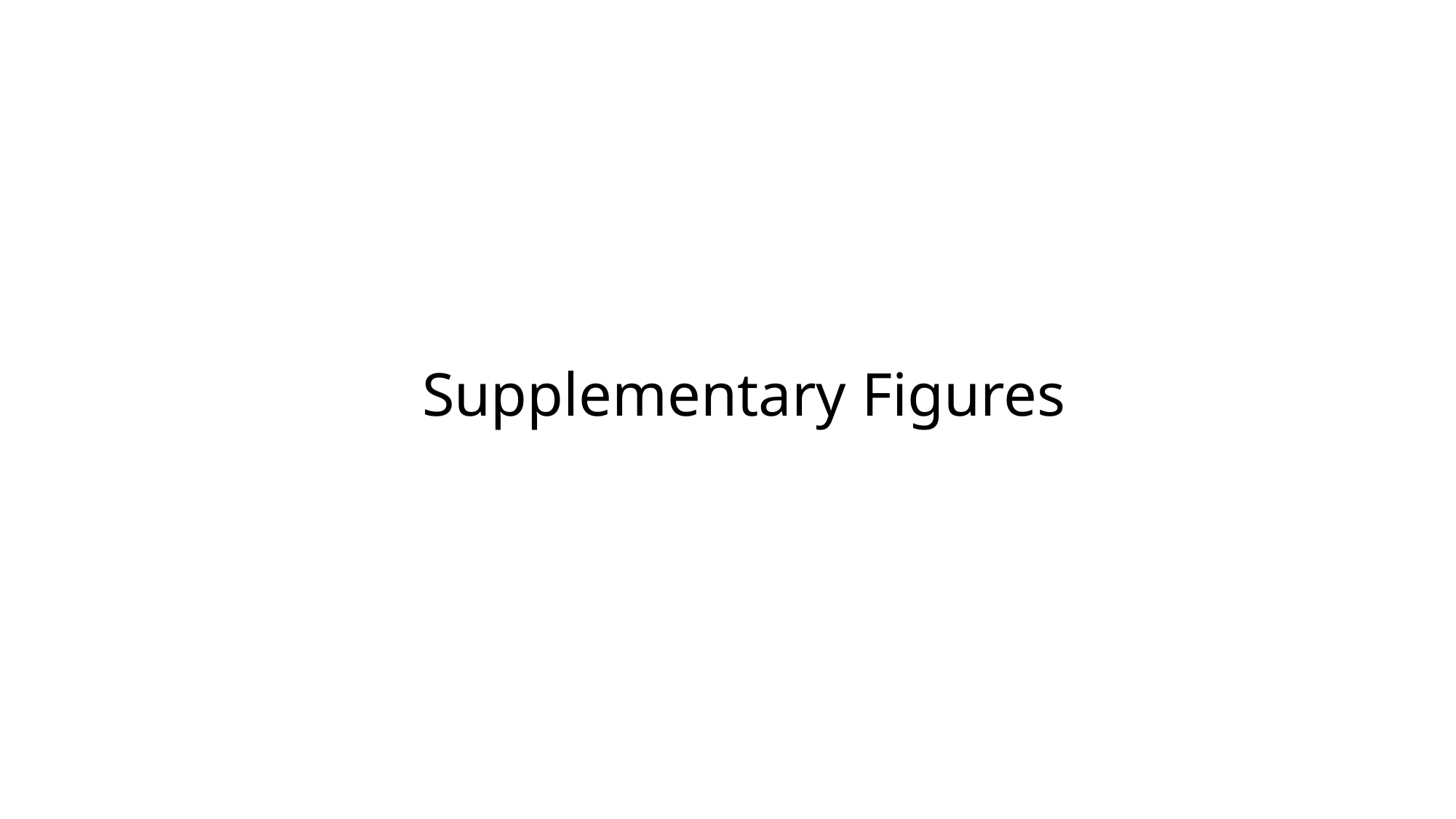

# Supplementary Figures

## Slide 2
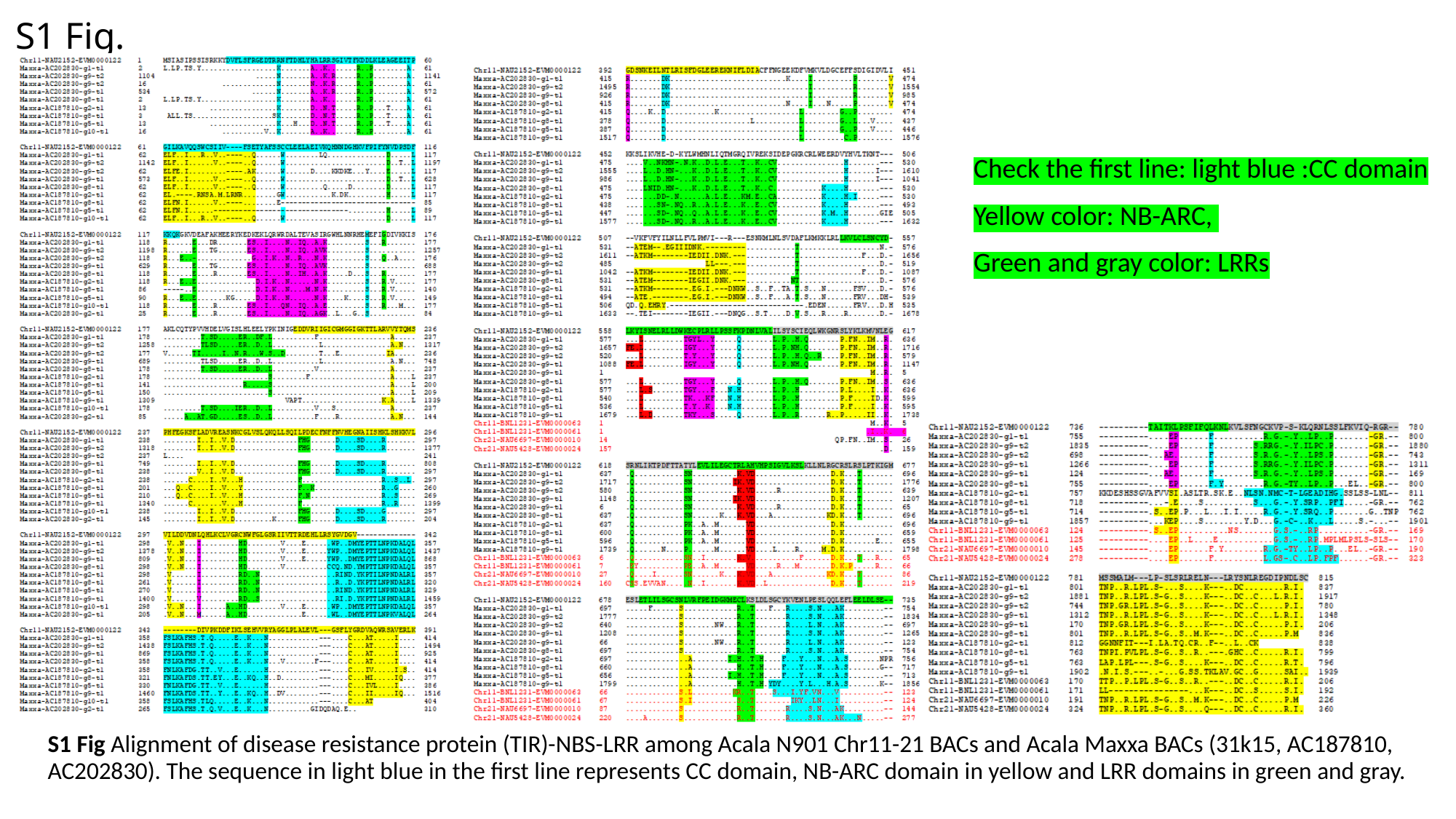

# S1 Fig.
Check the first line: light blue :CC domain
Yellow color: NB-ARC,
Green and gray color: LRRs
S1 Fig Alignment of disease resistance protein (TIR)-NBS-LRR among Acala N901 Chr11-21 BACs and Acala Maxxa BACs (31k15, AC187810, AC202830). The sequence in light blue in the first line represents CC domain, NB-ARC domain in yellow and LRR domains in green and gray.

## Slide 3
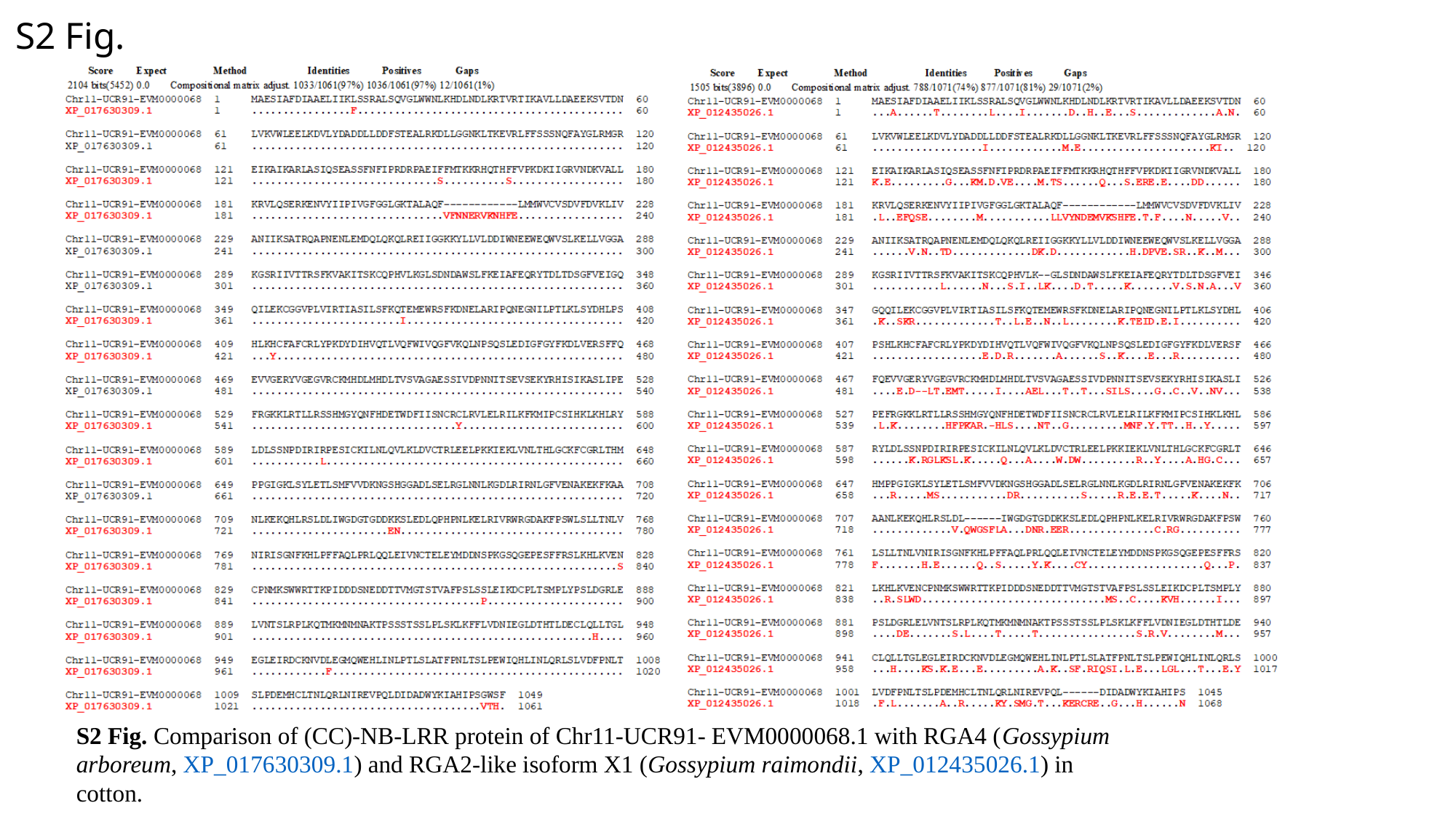

S2 Fig.
S2 Fig. Comparison of (CC)-NB-LRR protein of Chr11-UCR91- EVM0000068.1 with RGA4 (Gossypium arboreum, XP_017630309.1) and RGA2-like isoform X1 (Gossypium raimondii, XP_012435026.1) in cotton.

## Slide 4
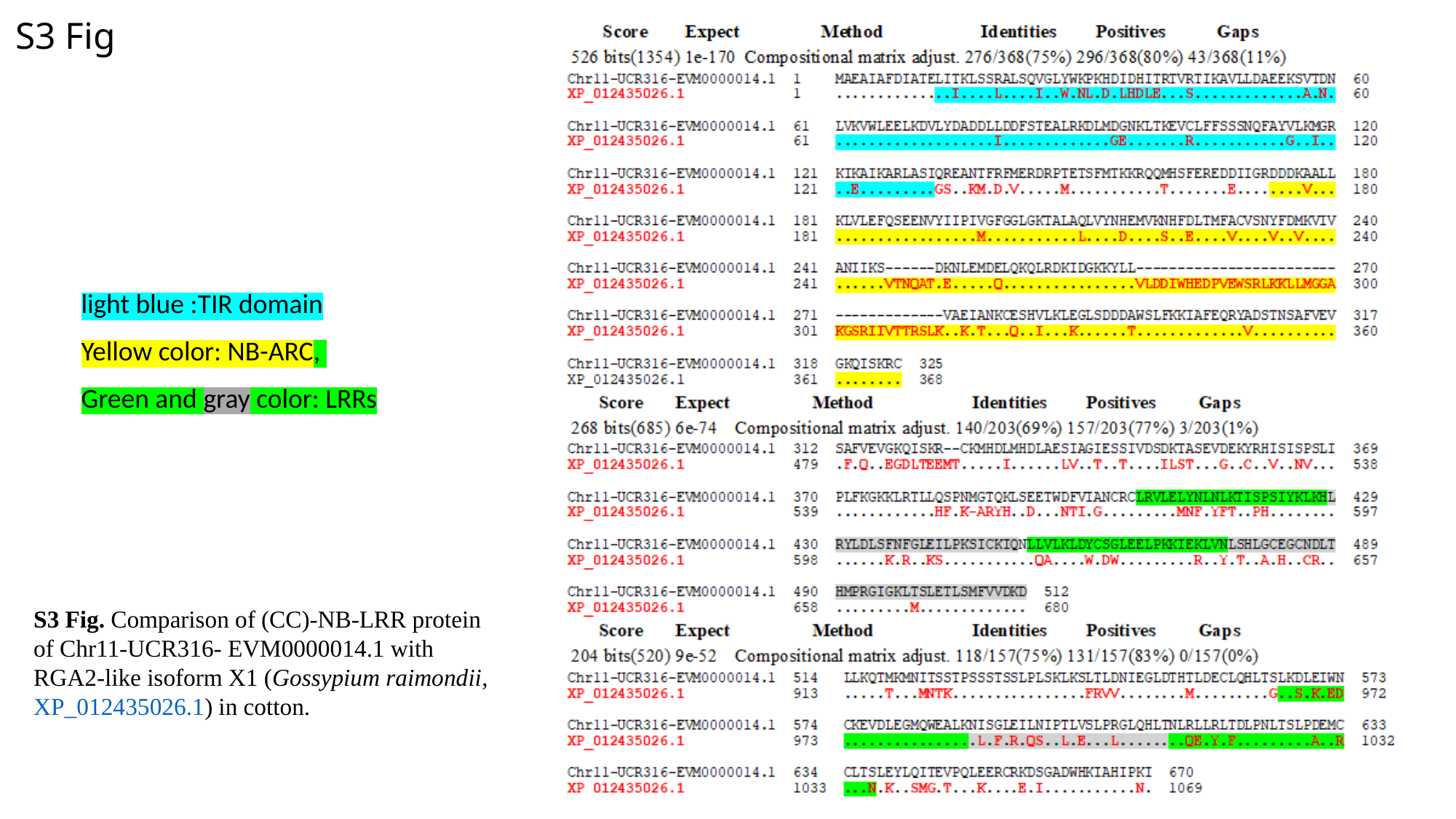

S3 Fig
light blue :TIR domain
Yellow color: NB-ARC,
Green and gray color: LRRs
S3 Fig. Comparison of (CC)-NB-LRR protein of Chr11-UCR316- EVM0000014.1 with RGA2-like isoform X1 (Gossypium raimondii, XP_012435026.1) in cotton.

## Slide 5
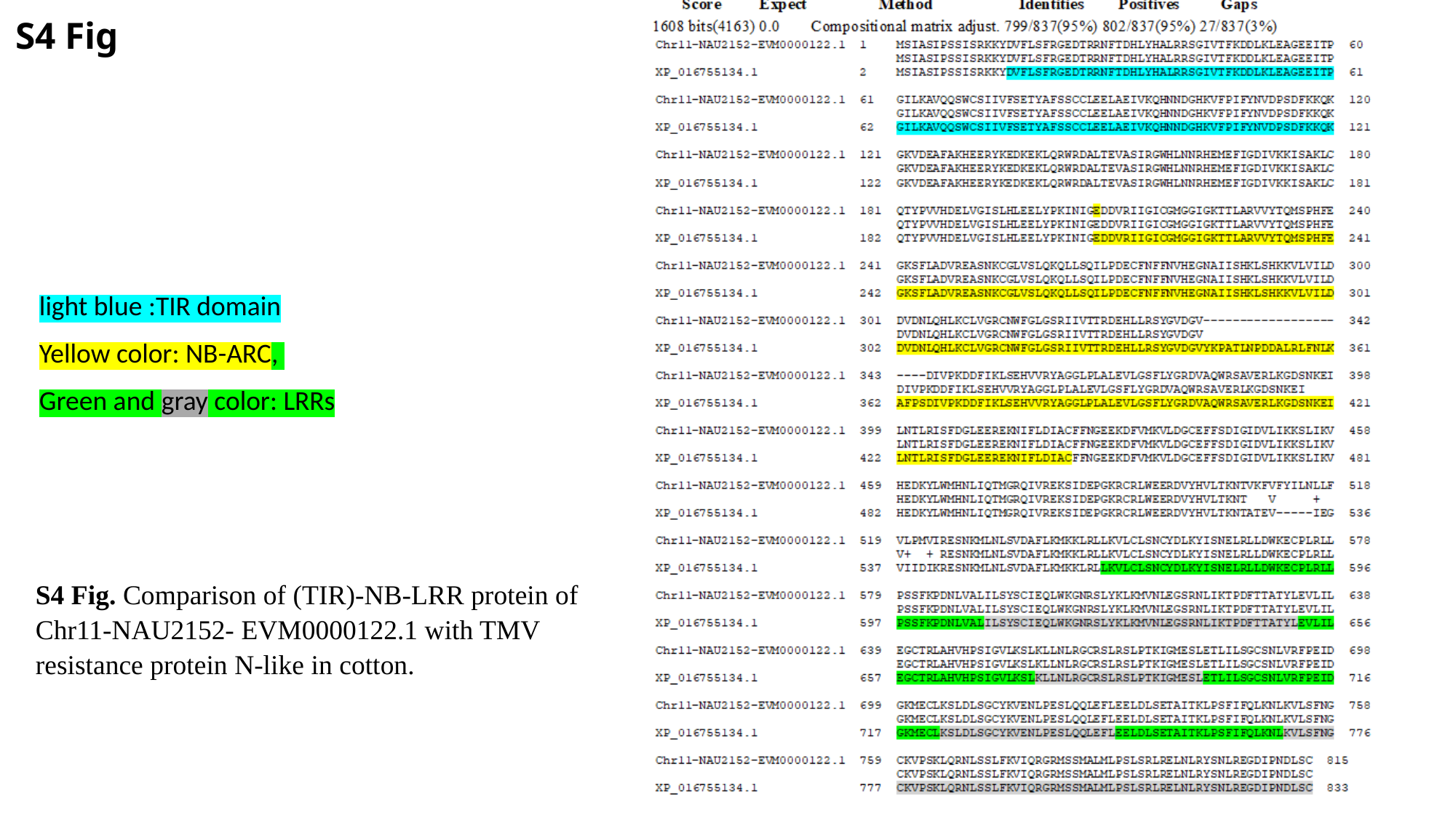

S4 Fig
light blue :TIR domain
Yellow color: NB-ARC,
Green and gray color: LRRs
S4 Fig. Comparison of (TIR)-NB-LRR protein of Chr11-NAU2152- EVM0000122.1 with TMV resistance protein N-like in cotton.

## Slide 6
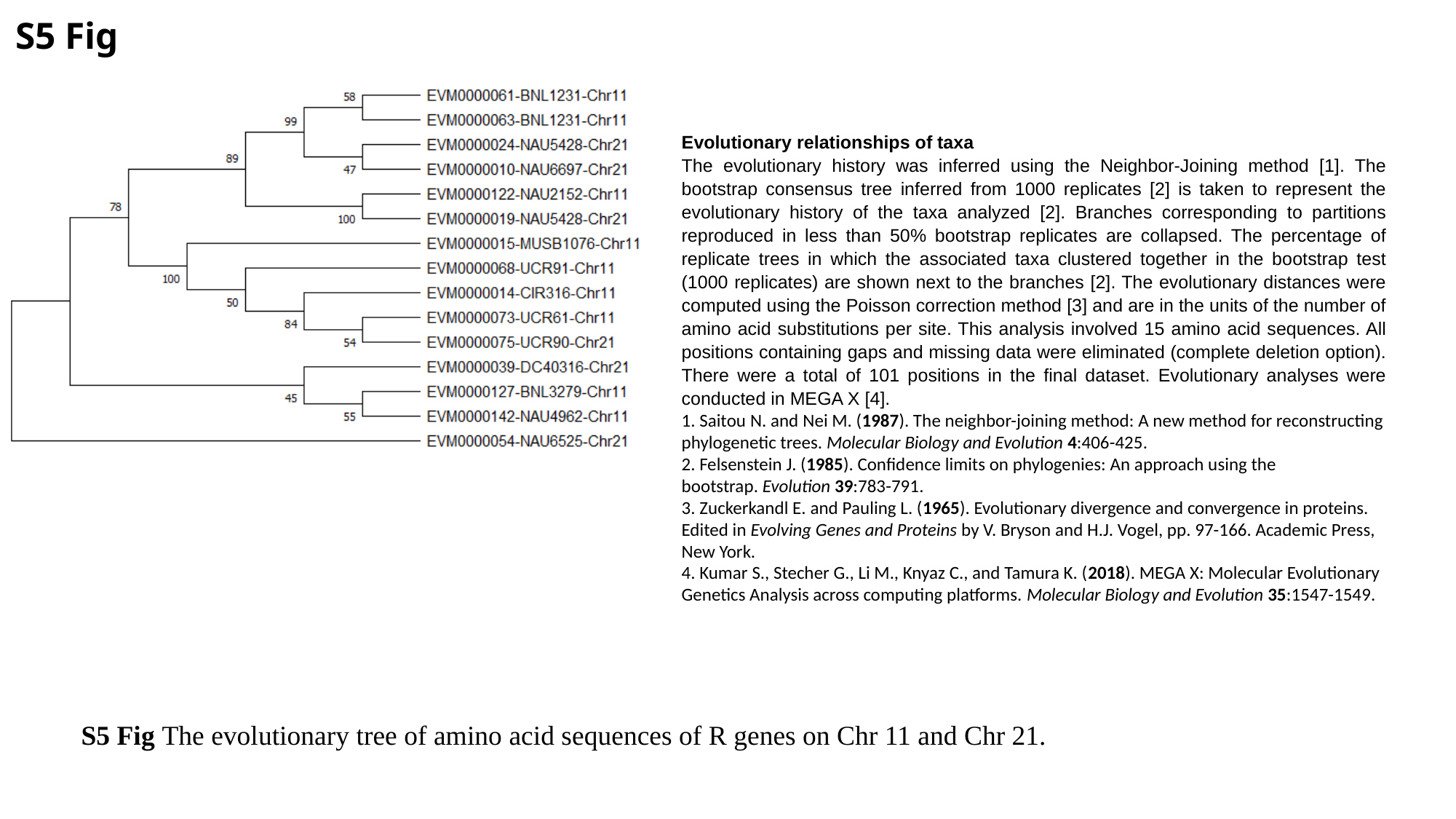

S5 Fig
Evolutionary relationships of taxa
The evolutionary history was inferred using the Neighbor-Joining method [1]. The bootstrap consensus tree inferred from 1000 replicates [2] is taken to represent the evolutionary history of the taxa analyzed [2]. Branches corresponding to partitions reproduced in less than 50% bootstrap replicates are collapsed. The percentage of replicate trees in which the associated taxa clustered together in the bootstrap test (1000 replicates) are shown next to the branches [2]. The evolutionary distances were computed using the Poisson correction method [3] and are in the units of the number of amino acid substitutions per site. This analysis involved 15 amino acid sequences. All positions containing gaps and missing data were eliminated (complete deletion option). There were a total of 101 positions in the final dataset. Evolutionary analyses were conducted in MEGA X [4].
1. Saitou N. and Nei M. (1987). The neighbor-joining method: A new method for reconstructing phylogenetic trees. Molecular Biology and Evolution 4:406-425.
2. Felsenstein J. (1985). Confidence limits on phylogenies: An approach using the bootstrap. Evolution 39:783-791.
3. Zuckerkandl E. and Pauling L. (1965). Evolutionary divergence and convergence in proteins. Edited in Evolving Genes and Proteins by V. Bryson and H.J. Vogel, pp. 97-166. Academic Press, New York.
4. Kumar S., Stecher G., Li M., Knyaz C., and Tamura K. (2018). MEGA X: Molecular Evolutionary Genetics Analysis across computing platforms. Molecular Biology and Evolution 35:1547-1549.
S5 Fig The evolutionary tree of amino acid sequences of R genes on Chr 11 and Chr 21.

## Slide 7
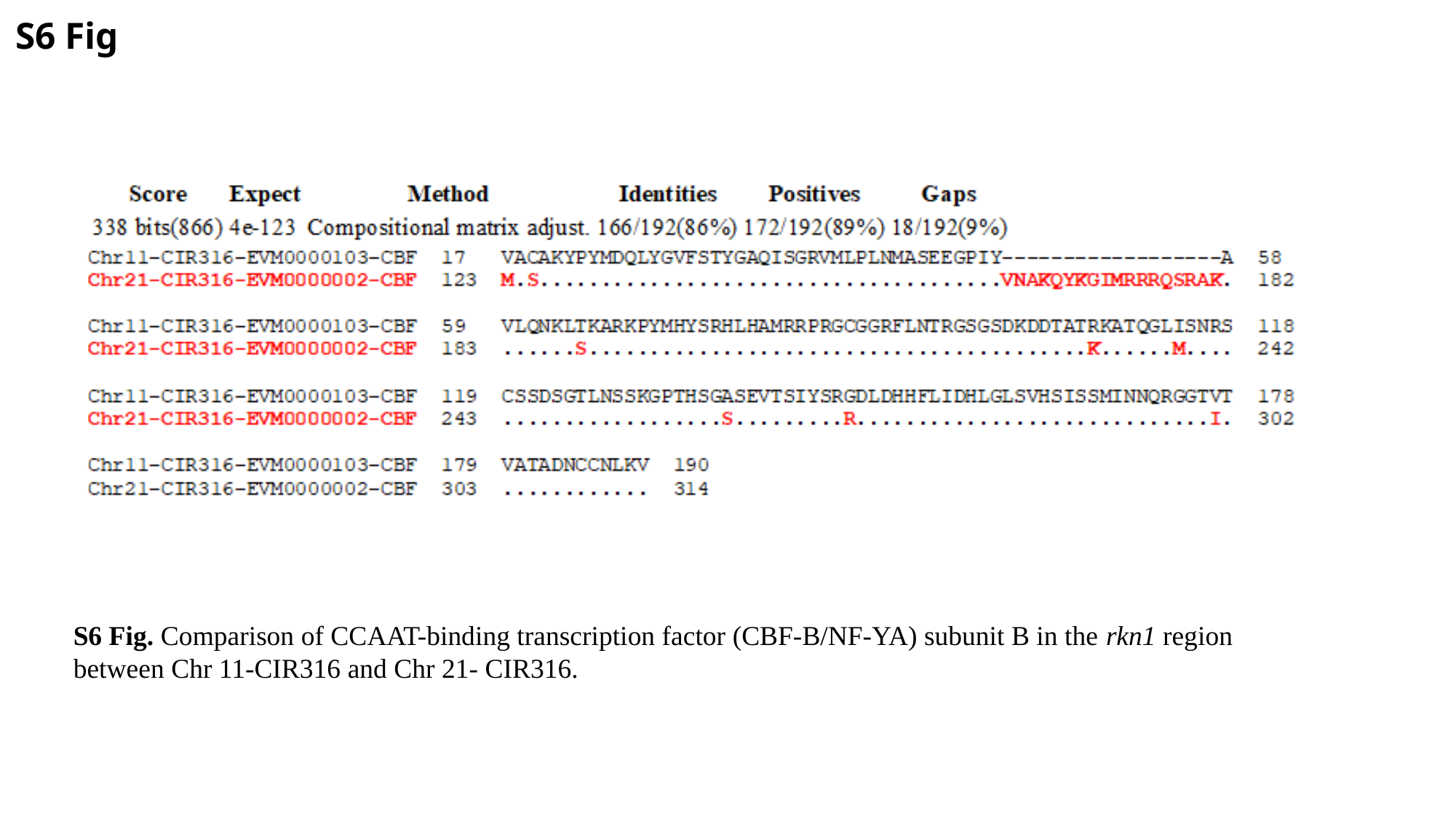

S6 Fig
S6 Fig. Comparison of CCAAT-binding transcription factor (CBF-B/NF-YA) subunit B in the rkn1 region between Chr 11-CIR316 and Chr 21- CIR316.

## Slide 8
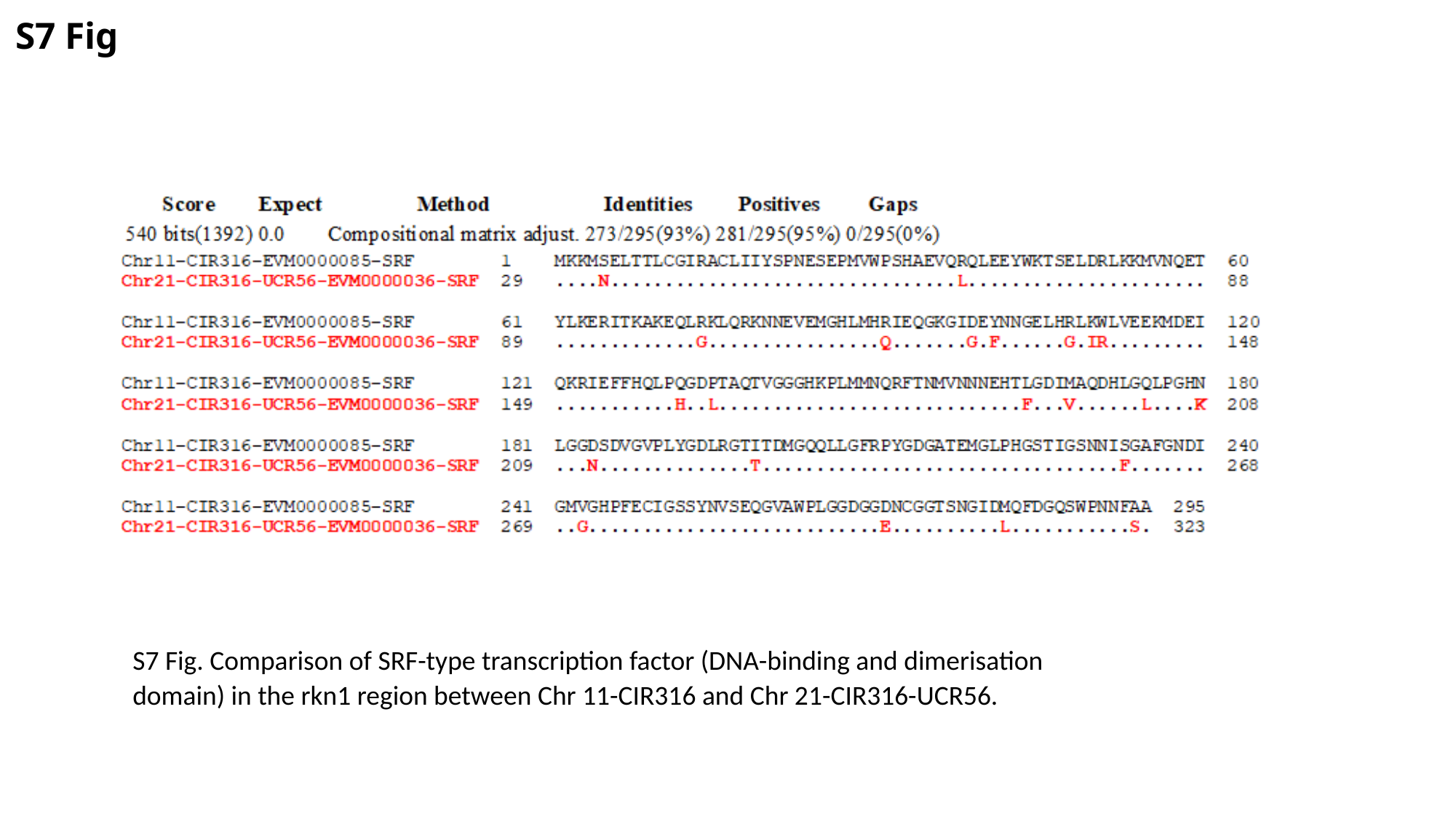

S7 Fig
S7 Fig. Comparison of SRF-type transcription factor (DNA-binding and dimerisation domain) in the rkn1 region between Chr 11-CIR316 and Chr 21-CIR316-UCR56.

## Slide 9
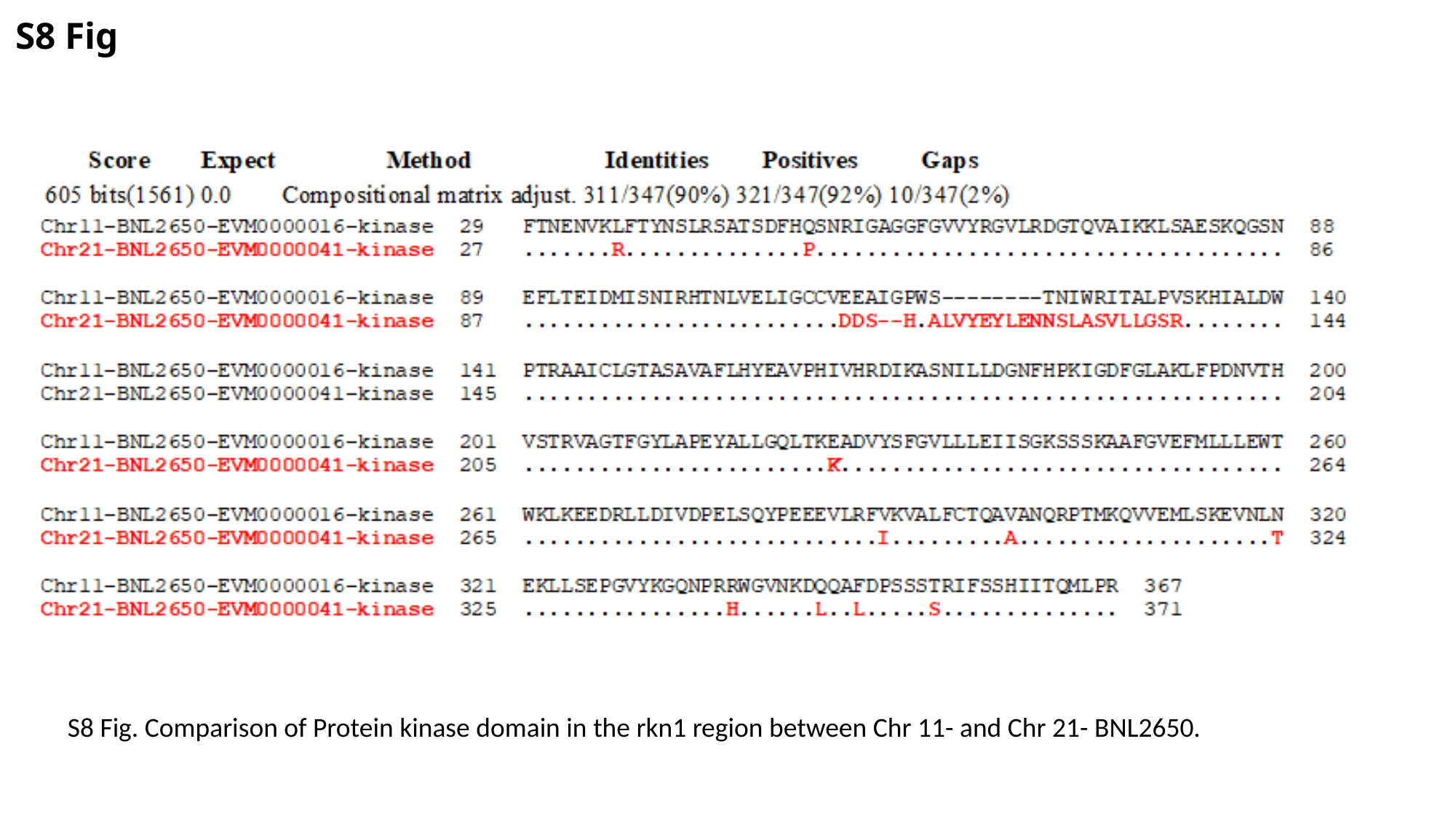

S8 Fig
S8 Fig. Comparison of Protein kinase domain in the rkn1 region between Chr 11- and Chr 21- BNL2650.

## Slide 10
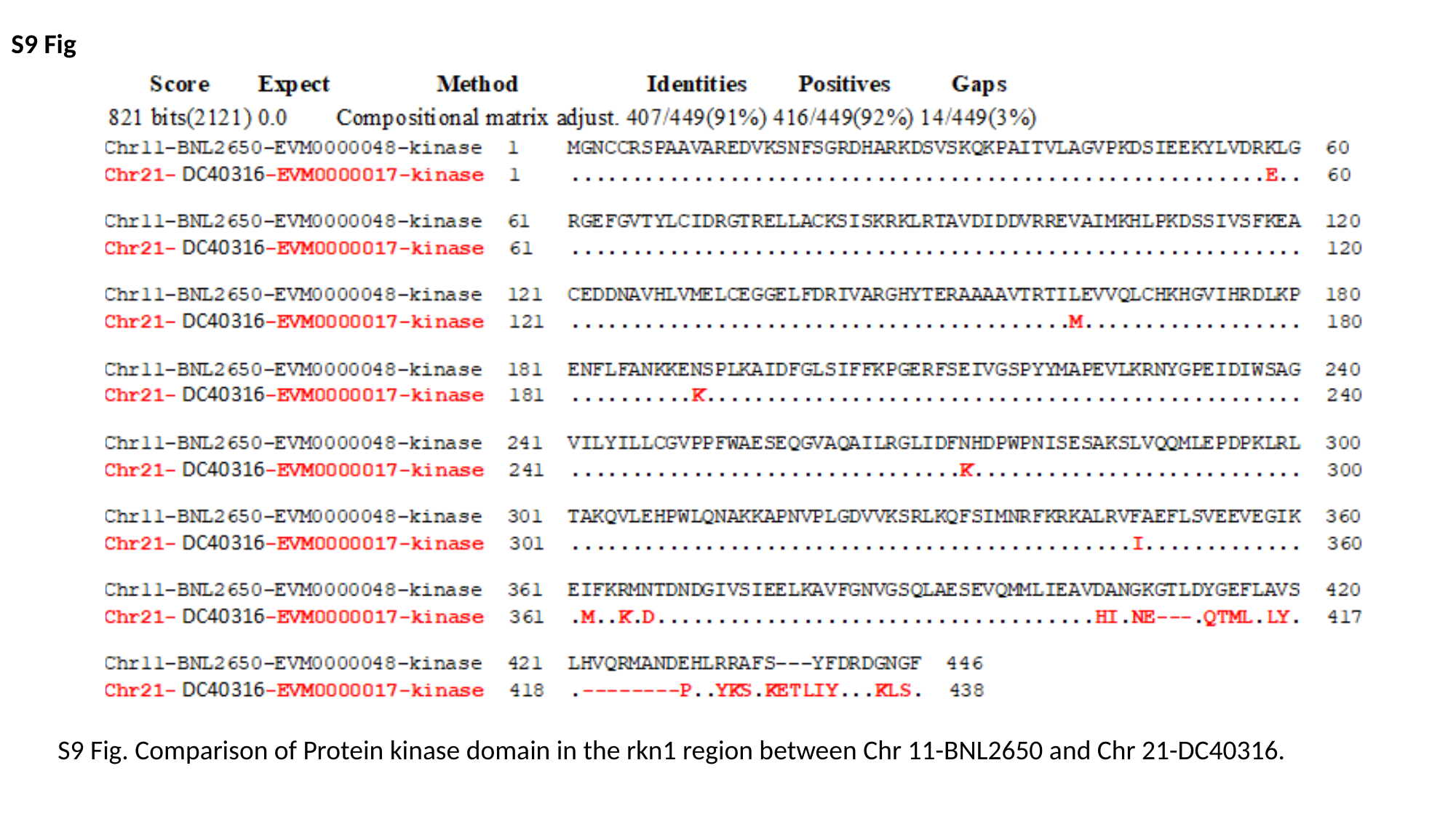

S9 Fig
S9 Fig. Comparison of Protein kinase domain in the rkn1 region between Chr 11-BNL2650 and Chr 21-DC40316.
